# Structural relationships among academic self-efficacy, academic performance, and career preparation behavior: the roles of value and cost as mediating variables

**DOI:** 10.3389/fpsyg.2025.1680088

**Published:** 2026-01-29

**Authors:** Yu Zhi, Cheon-woo Han

**Affiliations:** 1 Shanghai Normal University Tianhua College, Shanghai, China; 2 Department of Education, Keimyung University, Daegu, Republic of Korea

**Keywords:** academic performance, academic self-efficacy, career preparation behavior, China, cost, expectancy-value theory, higher education, value

## Abstract

**Introduction:**

This study examined how academic self-efficacy influences college students' academic performance and career preparation behavior, with value and cost acting as serial mediators.

**Methods:**

Based on social cognitive theory and expectancy-value theory, structural equation modeling was performed using data from 1,182 undergraduates at Chinese private universities.

**Results:**

Results showed that self-efficacy directly and indirectly predicted both outcomes. A value-to-cost serial mediation pathway was confirmed, indicating that higher self-efficacy increased value, lowered cost, and subsequently improved GPA and career preparation behavior. The model demonstrated a good fit.

**Discussion:**

The findings emphasize the importance of strengthening value while reducing costs, and provide both theoretical insights and practical strategies for improving academic and career outcomes in higher education.

## Introduction

In recent years, private universities have become an increasingly significant component of China's higher education system, accounting for nearly one-quarter of all undergraduate enrollment (Ministry of Education of the People's Republic of China, 2022). However, students enrolled in these institutions often face systemic disadvantages, including limited access to academic resources, weaker institutional reputation, and less effective career support services (Li and Morgan, 2008; Mok, 2016). As a result, their ability to succeed academically and prepare for the labor market has become a focal concern for educators and policymakers.

Academic performance and career preparation behavior are two vital outcomes of undergraduate education. While GPA is a widely accepted measure of academic achievement, career preparation behavior involves activities like exploring job opportunities, gaining career-related skills, and participating in internships or career counseling (Van Hooft et al., 2005; Sung et al., 2024). Evidence suggests that students attending private universities in China tend to face greater challenges in both academic performance and career preparation due to disparities in institutional resources and reputational advantages (Li and Morgan, 2008; Mok, 2016). This disparity underscores the importance of investigating the psychological factors that influence students' academic and career engagement.

Recent studies (2021-2025) further support the roles of self-efficacy, value, and cost in academic and career development. For example, Kim and Ra (2022) found that academic self-efficacy not only directly predicts academic performance but also promotes career preparation behavior by enhancing career decision-making self-efficacy (Kim and Ra, 2022). Meanwhile, Jiang Y. et al. (2020) demonstrated that perceived cost, particularly emotional cost, mediates the relationship between self-efficacy and academic avoidance among Chinese high school students (Jiang Y. et al., 2020). Additionally, Edwards and Dai (2023) highlighted the moderating role of task value in generational differences regarding self-efficacy and academic engagement (Edwards and Dai, 2023). These studies collectively emphasize the importance of integrating self-efficacy, value, and cost into a comprehensive model, especially in non-Western, high-pressure educational contexts.

Drawing on social cognitive theory (Bandura, 1997) and expectancy-value theory (Eccles and Wigfield, 2002), this study investigates three motivational constructs: academic self-efficacy, value, and cost. Although previous research has examined these variables separately, few have integrated them into a single explanatory model within non-elite institutional settings in East Asia. To fill this gap, we propose a serial mediation model, by examining how self-efficacy influences GPA and career preparation through value and cost. This framework not only clarifies the motivational dynamics driving students' engagement and future readiness in resource-constrained environments but also offers practical insights for supporting students in under-resourced higher education environments.

### Theoretical framework

Much of the early research on academic and career outcomes focused on cognitive indicators such as academic ability, prior achievement, and socioeconomic background (Robbins et al., 2004; Pascarella and Terenzini, 2005). However, recent scholarship underscores the role of non-cognitive factors in shaping students' academic performance and career development (Heckman et al., 2006; Richardson et al., 2012). Grounded in social cognitive theory (Bandura, 1986) and expectancy-value theory (Eccles et al., 1983), this study focuses on three key motivational constructs, academic self-efficacy, value, and cost, that jointly influence students' engagement, persistence, and future readiness. These constructs have been empirically validated across educational contexts and are increasingly recognized for their predictive value in understanding both academic success and career preparation (Eccles and Wigfield, 2002; Flake et al., 2015; Jiang Z. et al., 2020). By integrating these variables into a unified framework, the study aims to clarify how students' motivational beliefs translate into concrete academic and vocational behaviors, especially within the high-pressure context of Chinese higher education (Gao et al., 2020; Wang and Liu, 2022).

### Academic self-efficacy

Rooted in social cognitive theory, self-efficacy refers to individuals' beliefs in their ability to organize and execute actions to achieve specific goals (Bandura, 1977). In academic contexts, academic self-efficacy reflects students' perceived competence in managing learning and achieving educational success (Bandura, 1993; Schunk, 1989), representing their confidence in their academic capabilities (Kandemir, 2014).

Academic self-efficacy is positively associated with value, academic performance, and career preparation, while negatively related to cost (Wigfield and Eccles, 2000; Eccles and Wigfield, 2002; Kim and Ra, 2022). Students with strong self-efficacy are more likely to view academic tasks as meaningful and aligned with future goals, enhancing value and sustaining motivation (Wigfield and Eccles, 2000). At the same time, they perceive academic demands as manageable, which reduces cost and supports greater persistence and engagement (Eccles and Wigfield, 2002). This confidence promotes effective learning strategies, better academic outcomes, and constructive responses to setbacks (Zimmerman and Martinez-Pons, 1990).

Moreover, academic self-efficacy predicts proactive career behaviors by fostering goal clarity, decision-making confidence, and participation in career-related activities (Lent et al., 2000). Empirical studies across diverse contexts confirm these links, emphasizing academic self-efficacy's central role in shaping students' motivation, learning outcomes, and future planning (Kim and Ra, 2022).

### Value

In expectancy-value theory, value is central to understanding why students choose to engage in academic and career-related activities (Eccles and Wigfield, 2002). It encompasses perceptions of how important, interesting, or useful a task is, typically categorized into attainment, intrinsic, and utility value (Wigfield and Cambria, 2010). When students find tasks meaningful, enjoyable, or relevant to future goals, they are more likely to invest sustained effort and employ deeper learning strategies (Gaspard et al., 2015; Perez et al., 2014).

Empirical evidence shows that value is a strong predictor of academic performance. Students who perceive higher value in academic tasks tend to show greater motivation and persistence, which contributes to improved GPA (Edwards and Dai, 2023). These effects are further enhanced when value is aligned with personal goals or career aspirations. Moreover, value not only supports academic achievement but also predicts career preparation behaviors. Utility and attainment value, in particular, drive participation in career planning, internships, and skill development by emphasizing the long-term benefits of current learning (Durik et al., 2006; Lee et al., 2022).

While value has been widely studied, its combined role with self-efficacy and cost remains underexplored, especially in non-Western contexts. Recognizing the motivational function of value across academic and vocational domains is essential for understanding how students navigate educational demands and prepare for future transitions.

### Cost

Although cost was initially conceptualized as part of the broader value construct, recent studies emphasize its distinct psychological function in motivation theory. Unlike positively framed value components, cost captures the negative expectations associated with academic engagement, including time, effort, emotional stress, and potential failure (Flake et al., 2015; Perez et al., 2014). These costs are shown to reduce engagement even when value and efficacy are high (Barron and Hulleman, 2015).

Research has consistently found that cost exerts a negative influence on academic behavior. Emotional cost, such as anxiety and fear of failure, contributes to avoidance and procrastination, while opportunity cost discourages students from investing effort when academic tasks interfere with more rewarding alternatives (Barron and Hulleman, 2015). Ego cost may further hinder participation by threatening self-worth or competence beliefs, particularly in high-stakes academic settings (Perez et al., 2014).

Cost also affects students' willingness to engage in career preparation activities. Drawing on conservation of resources theory (Hobfoll, 1989), students may avoid cognitively or emotionally demanding tasks like internships or portfolio building to preserve their psychological resources. In both academic and career domains, cost has been found to moderate or mediate motivational processes, especially under high-pressure environments such as Chinese higher education (Jiang Y. et al., 2020). These findings underscore the value of treating cost as an independent construct in models of student motivation and behavior.

### Academic performance

Academic performance serves as a key indicator of students' academic progress and is commonly assessed through measures such as exam scores, course grades, and cumulative GPA. Among these, GPA is widely used due to its ability to reflect academic engagement and outcomes across multiple semesters (Pascarella and Terenzini, 2005). While early definitions emphasized test scores and rankings, contemporary understandings incorporate both cognitive outcomes and broader skills relevant to lifelong learning, including critical thinking, collaboration, and self-regulated learning strategies (Facione, 2011; Mentkowski and Doherty, 1984).

Research on academic performance has traditionally focused on cognitive factors such as intelligence and prior achievement. However, recent studies highlight the role of motivational and emotional constructs, particularly academic self-efficacy, in shaping learning outcomes (Bandura, 1997; Pajares, 1996). Students with higher self-efficacy are more persistent and adopt effective learning strategies, leading to improved academic performance.

This study adopts a narrow definition of academic performance, using GPA as the primary indicator. This approach aligns with prior research and provides an objective, standardized measure suitable for quantitative analysis (Richardson et al., 2012). While recognizing the broader dimensions of student development, GPA is used here to operationalize academic success within a measurable and comparable framework.

### Career preparation behavior

Career preparation behavior refers to concrete, goal-oriented activities that support individuals in exploring, planning, and pursuing their future careers. Rather than focusing solely on cognitive or attitudinal components, it emphasizes observable actions such as acquiring job-related skills, engaging in career exploration, and preparing application materials (Niu, 2010; Kim and Kim, 1997).

Empirical research has identified multiple dimensions of career preparation behavior, ranging from early-stage exploration (e.g., gathering career information, reflecting on goals) to active engagement (e.g., resume writing, internships, certification exams) (Sung et al., 2024; Van Hooft et al., 2021). These behaviors are often associated with higher levels of career decision-making self-efficacy, proactive planning, and job readiness (Lee et al., 2022; Van Hooft et al., 2021). In particular, skill development, work experience, and participation in career services have shown strong predictive value for employment outcomes (Sagen et al., 2000; Skorikov, 2007).

Theoretical frameworks such as social cognitive career theory and expectancy-value theory suggest that career preparation behavior is shaped by students' beliefs about their competence, perceived value of career actions, and the cost of engagement (Lent et al., 1994; Eccles and Wigfield, 2002). In high-pressure environments like China, where academic and familial expectations are high, career preparation behavior offers a practical lens to assess students' readiness and engagement beyond attitudes or intentions (Li, 2018).

### The present study

**Figure 1:**
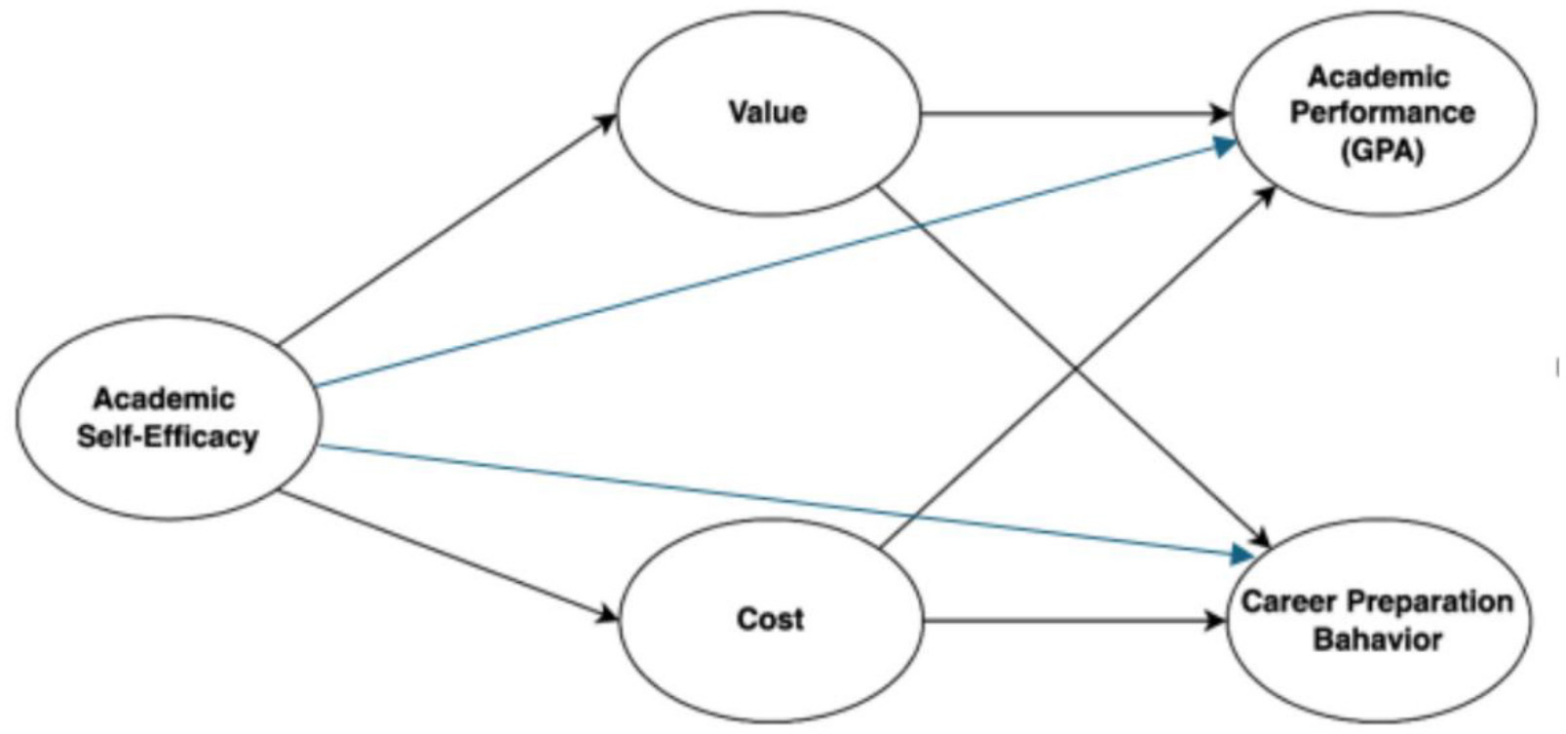
Hypothesized structural model.

Situated within the landscape of Chinese higher education, this study explores the combined effects of academic self-efficacy, value, and cost on undergraduates' academic performance and career preparation behavior. The outcomes aim to inform both theoretical discourse and practical strategies for enhancing university-level career development programs. Based on the theoretical framework and prior empirical findings, we propose the following directional hypotheses (Figure 1):

H1: Academic self-efficacy positively predicts academic performance (GPA).
H2: Academic self-efficacy positively predicts career preparation behavior.
H3: Value mediates the relationship between academic self-efficacy and academic performance (GPA).
H4: Value mediates the relationship between academic self-efficacy and career preparation behavior.
H5: Cost mediates the relationship between academic self-efficacy and academic performance (GPA).
H6: Cost mediates the relationship between academic self-efficacy and career preparation behavior.
H7: Value and cost jointly serve as serial mediators in the relationship between academic self-efficacy and academic performance (GPA).
H8: Value and cost jointly serve as serial mediators in the relationship between academic self-efficacy and career preparation behavior.

## Method

### Participants

A total of 1,231 undergraduate students (834 females, 67.75%; 397 males, 32.25%) from 17 private universities in Shanghai participated in this study. The average age of participants was 20.3 years (SD = 1.2), with sophomores averaging 19.8 years (SD = 0.9), juniors 20.5 years (SD = 1.1), and seniors 21.1 years (SD = 1.0) (see Table 1). Participants were selected using a stratified random sampling method. The sampling frame consisted of all sophomore, junior, and senior students from the 17 institutions. Students were stratified by academic year and institution. Within each stratum, a random number generator was used to select participants from student registration lists provided by university administration offices. Freshmen were excluded due to the lack of GPA records. After data cleaning to remove incomplete or inconsistent responses, 1,182 valid cases were retained. This sampling strategy ensured sufficient representation across academic levels and institutions to address the research objectives.

**Table 1:** Demographic characteristics and age distribution of participants.

**Variable**	**Category**	***n***	**Percentage (%)**	**Age (M ±SD)**
Total	-	1,231	100.00%	20.3 ± 1.2
Gender	Male	397	32.25%	-
	Female	834	67.75%	-
Grade	Sophomore	384	31.20%	19.8 ± 0.9
	Junior	356	28.90%	20.5 ± 1.1
	Senior	473	38.40%	21.1 ± 1.0

### Measures

#### Academic self-efficacy

Academic Self-Efficacy was assessed using the nine-item Self-Efficacy Scale from the Motivated Strategies for Learning Questionnaire (MSLQ) (Pintrich et al., 1991), adapted for Chinese contexts from the MSLQ-CV, which has shown good reliability and valid factor structure in Chinese settings (Lee et al., 2010; Rao and Sachs, 1999). Items measured students' academic competence (e.g., “I expect to do well in this class”) and confidence in positive outcomes (e.g., “I believe I will get a good grade”). Responses were provided on a 5-point Likert scale from 1 (“not true at all”) to 5 (“very true”). The scale in this study demonstrated excellent internal consistency with a Cronbach's α of 0.910.

#### Value

This study employed the Task Value Scale from the Motivated Strategies for Learning Questionnaire (MSLQ) to assess participants' perceptions of value (Pintrich et al., 1991). The scale consists of four items measuring how important and useful students perceive academic tasks to be. Items 1 and 2 measure utility value, while items 3 and 4 measure attainment value. Although brief, this scale has demonstrated strong psychometric properties in Chinese student samples, with established convergent and discriminant validity (Rao and Sachs, 1999; Lee et al., 2010). No items with factor loadings below 0.7 were identified during pilot testing, indicating that the scale effectively captures core dimensions of task value despite its conciseness. The “α if item deleted” values are reported to demonstrate that each item contributes positively to overall scale reliability and that removing any item would not substantially improve Cronbach's alpha. Responses were given on a 5-point Likert scale from 1 (“not true at all”) to 5 (“very true”). In this study, the reliability of the value scale was tested. The “α if item deleted” values were as follows: α = 0.951 (Q1), α = 0.962 (Q2), α = 0.947 (Q3), and α = 0.948 (Q4). The scale demonstrated high overall internal consistency, with α = 0.886, indicating high internal consistency and reliability. Therefore, the questionnaire design is deemed reasonable and highly reliable.

#### Cost

This study employed a shortened version of the Cost Scale, originally developed to assess participants' perceived costs in an academic context (Jiang Y. et al., 2020). Specifically, Items 1 and 2 measure opportunity cost, Items 3 and 4 measure ego cost, and Items 5 and 6 measure emotional cost. This abbreviated version has been validated in multiple Chinese educational studies, demonstrating good reliability and validity while minimizing respondent fatigue in cross-institutional surveys. Participants responded to the items using a 5-point Likert scale, ranging from 1 (“not true at all”) to 5 (“very true”). In this study, the reliability of the Cost Scale was tested. The “α if item deleted” values for each item were as follows: α = 0.931 (Q1), α = 0.930 (Q2), α = 0.925 (Q3), α = 0.923 (Q4), α = 0.925 (Q5), and α = 0.929 (Q6). These values indicate that each item contributed positively to the internal consistency of the scale. Additionally, the overall internal consistency of the scale was found to be high, with a Cronbach's α value of 0.877. Based on these findings, the questionnaire design is considered appropriate and exhibits strong internal reliability.

#### Academic performance

Students' academic performance was measured by their GPA on a 5-point scale. This method is widely adopted in many Chinese universities (Liu et al., 2025). GPA data were obtained from university records with the assistance of student affairs offices, ensuring accuracy and consistency across institutions.

#### Career preparation behavior

This study used the Career Preparation Behavior Scale, adapted to evaluate participants' career preparation behavior and decision-making (Sung et al., 2024). The scale includes five items, each rated on a 5-point Likert scale from 1 (“not true at all”) to 5 (“very true”). The scale evaluates dimensions such as exploring career options, researching qualifications and materials, seeking advice from reliable sources, and purchasing relevant materials. Additionally, it assesses the career decision-making level, which involves activities like taking assessments, gathering information about various occupations, and seeking help from others. The scale demonstrated strong reliability, with α = 0.901, indicating high internal consistency. This tool was adopted to measure participants' engagement in career-related behaviors and decision-making processes.

#### Procedure

Data collection was conducted between November and December 2024. The research team obtained approval from student affairs departments at 17 private universities in Shanghai. An online questionnaire, administered via Questionnaire Star, was distributed using a QR code to eligible students, who were informed of the study's purpose and confidentiality assurances. A total of 1,400 questionnaires were distributed, with 1,231 returned, yielding a response rate of 87.9%. After data cleaning, 1,182 valid responses were retained, resulting in an effective response rate of 84.4%. The survey took approximately 5–8 mins to complete and included 27 items assessing academic self-efficacy, value, cost, career preparation behavior, and demographic variables. A total of 1,231 responses were collected. To ensure data quality, responses were screened following established self-report instrument protocols (Podsakoff et al., 2003). Cases with irregular response patterns or implausibly short completion times were excluded, resulting in 1,182 valid responses. Data were analyzed using SPSS 26.0 and AMOS 25.0, including descriptive statistics, reliability checks, and structural equation modeling.

#### Analytic plan

Preliminary analyses were conducted using SPSS 26.0 and AMOS 25.0 to examine the psychometric properties and structural relationships among the study variables. The questionnaire consisted of 24 items across four latent constructs: academic self-efficacy (9 items), value (4 items), cost (6 items), and career preparation behavior (5 items), each rated on a 5-point Likert scale. To assess potential common method variance, Harman's single-factor test was conducted. The unrotated factor solution revealed that the first factor accounted for 32.7% of the variance, below the 50% threshold, suggesting that common method bias was not a major concern. Cronbach's α coefficients indicated strong internal consistency for all subscales (α = 0.877 to 0.910), with the overall scale reliability at 0.890

Descriptive statistics and Pearson correlation analyses were first performed to assess basic relationships among academic self-efficacy, value, cost, GPA, and career preparation behavior. To validate the measurement model, Confirmatory Factor Analysis (CFA) was conducted. An initial CFA model specifying four correlated factors was tested. After allowing correlations between error terms of items within the same subscale based on modification indices, the final CFA model showed improved fit. Model fit was evaluated using multiple indices: χ^2^*/df* (<3), CFI and TLI (≥0.90), and RMSEA and SRMR ( ≤ 0.08), following established criteria (Kline, 2023).

Structural Equation Modeling (SEM) was employed to test the hypothesized model and mediation effects. SEM enables simultaneous estimation of latent variables and structural paths while accounting for measurement error, making it suitable for examining complex theoretical models in educational research (Byrne, 2013; Kline, 2023). All mediation analyses, including direct, indirect, and serial mediation effects, were conducted within the SEM framework in AMOS using the bootstrapping method with 5,000 resamples. Indirect effects were considered significant if the interval did not include zero (Preacher and Hayes, 2008; Hayes, 2017). Overall, SEM offered a robust framework to evaluate both direct and indirect effects, ensuring comprehensive model testing.

## Results

### Preliminary analyses

All statistical analyses were conducted using SPSS 26.0 and AMOS 25.0. The study retained 1,182 valid student responses. Descriptive statistics were first performed to examine distribution characteristics of key variables: academic self-efficacy, value (utility and attainment), cost (opportunity, ego, emotional), career preparation behavior, and academic performance (GPA). As shown in Table 2, mean scores ranged from 3.456 to 3.769, and standard deviations from 0.677 to 0.910. Skewness and kurtosis values confirmed that all variables were approximately normally distributed.

**Table 2:** Descriptive statistics of study variables.

**Variables**	**Sub-scales**	***M***	***SD***	**Skewness**	**Kurtosis**
Academic Self-Efficacy		3.678	0.677	−0.013	−0.542
Value	Utility value	3.769	0.738	−0.883	1.550
	Attainment value	3.756	0.711	−0.911	1.974
	Total	3.763	0.678	1.130	2.226
Cost	OPC	3.476	0.897	0.142	−0.878
	EGC	3.456	0.910	0.119	−0.892
	EMC	3.465	0.907	0.136	−0.884
	Total	3.466	0.811	0.080	−0.918
Career Preparation Behavior		3.644	0.726	−0.026	−0.014
Academic Performance (GPA)		3.680	0.805	−0.393	0.250

OPC, opportunity cost; EGC, ego cost; EMC, emotional cost.

### Correlations among variables

Pearson correlations were computed to explore relationships among variables. As presented in Table 3, academic self-efficacy positively correlated with value (*r* = 0.228–0.202), career preparation behavior (*r* = 0.341), and GPA (*r* = 0.305), and negatively correlated with cost (*r* = −0.402 to −0.428). Cost was negatively correlated with both career preparation behavior and GPA, supporting theoretical expectations.

**Table 3:** Correlation matrix.

**Variables**	**AS**	**UV**	**AV**	**OPC**	**EGC**	**EMC**	**CPB**	**GPA**
AS	1							
**Value**								
UV	0.228^**^	1						
AV	0.202^**^	0.753^**^	1					
**Cost**								
OPC	−0.427^**^	−0.401^**^	−0.388^**^	1				
EGC	−0.428^**^	−0.411^**^	−0.403^**^	0.702^**^	1			
EMC	−0.402^**^	−0.393^**^	−0.368^**^	0.706^**^	0.709^**^	1		
CPB	0.341^**^	0.364^**^	0.365^**^	−0.448^**^	−0.490^**^	−0.464^**^	1	
**Academic performance**								
GPA	0.305^**^	0.268^**^	0.298^**^	−0.346^**^	−0.333^**^	−0.360^**^	0.516^**^	1

AS, academic self-efficacy; UV, utility value; AV, attainment value; OPC, opportunity cost; EGC, ego cost; EMC, emotional cost; CPB, career preparation behavior; GPA, grade point average (academic performance); ^**^p < 0.01.

### Collinearity check

Given the high correlations among psychological cost dimensions (OPC, EGC, EMC; *r* = 0.702~0.709), variance inflation factors (VIFs) were calculated to assess multicollinearity. All VIF values were below 3, indicating that multicollinearity did not unduly influence parameter estimates.

### Confirmatory factor analysis

The final CFA model, modified from an initial four-factor model, showed good fit: χ^2^*/df* = 3.781, RMSEA = 0.049, SRMR = 0.022, CFI = 0.958, TLI =0.953 (Table 4). Standardized loadings exceeded 0.70, confirming convergent validity. Correlations among latent variables supported theoretical relationships.

**Table 4:** Model fit indices for the final CFA model.

**Model**	***χ^2^(p)***	***df***	***χ^2^/df***	**RMSEA**	**SRMR**	**CFI**	**TLI**
Measurement model	930.072	246	3.781	0.049	0.022	0.958	0.953
Structured model	930.072	246	3.781	0.049	0.022	0.958	0.953
Good fit index	*p* < 0.001		<3	<0.080	<0.080	>0.900	>0.900
Final model	930.072	246	3.781	0.049	0.022	0.958	0.953

χ^2^ = chi-square; df, degrees of freedom; RMSEA, root mean square error of approximation; SRMR, standardized root means square residual; CFI, comparative fit index; TLI, Tucker–Lewis's index. *p* < 0.001.

### Structural Equation Modeling (SEM)

**Figure 2:**
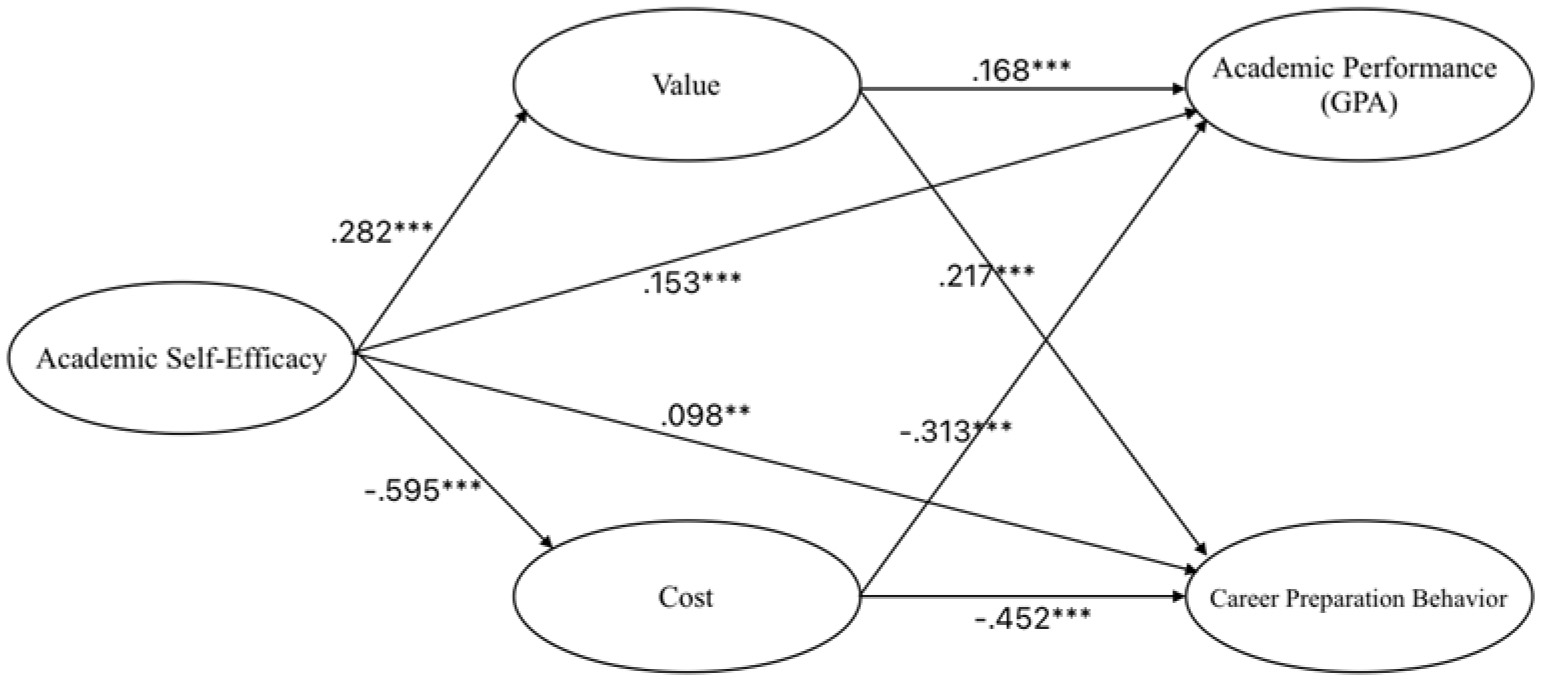
Path diagram of final SEM model. The asterisks denote statistical significance levels: **p < 0.01, ***p < 0.001.

The structural model demonstrated good fit and confirmed all hypothesized paths (Figure 2; Table 5). Academic self-efficacy positively predicted value (β = 0.282), GPA (β = 0.153), and career preparation behavior (β = 0.098), and negatively predicted cost (β = −0.595). Value positively affected GPA (β = 0.168) and career preparation behavior (β = 0.217), while cost negatively predicted both GPA (β = −0.313) and career preparation behavior (β = −0.452).

**Table 5:** Standardized path coefficients.

**Path**	**Standardized coefficient**	***p* value**	**Standard error**
Academic self-efficacy → value	0.282	^***^	0.032
Academic self-efficacy → cost	−0.595	^***^	0.038
Academic self-efficacy → academic performance (GPA)	0.153	^***^	0.041
Academic self-efficacy → career preparation behavior	0.098	^***^	0.038
Value → academic performance (GPA)	0.168	^***^	0.034
Value → career preparation behavior	0.217	^***^	0.031
Cost → academic performance (GPA)	−0.313	^***^	0.037
Cost → career preparation behavior	−0.452	^***^	0.036

^***^p < 0.001.

Although the direct effect of self-efficacy on career preparation behavior was relatively small (β = 0.098), indirect effects through value and cost were significant (see Table 5), suggesting that self-efficacy primarily influences career preparation via enhancing value perceptions and reducing cost perceptions.

### Mediation effects

Bootstrapping with 5,000 resamples within the SEM framework revealed significant indirect associations of academic self-efficacy on GPA and career preparation behavior via value and cost. Confidence intervals for all indirect paths excluded zero (Table 6), indicating significant partial mediation.

**Table 6:** Indirect effects via bootstrapping.

**Path**	**Original sample**	**Sample mean**	**Standard error**	**95% Cl**	
				**2.50%**	**97.50%**
Academic self-efficacy → value → academic performance (GPA)	0.040	0.041	0.001	0.015	0.064
Academic self-efficacy → cost → academic performance (GPA)	0.159	0.157	0.002	0.120	0.209
Academic self-efficacy → value → career preparation behavior	0.056	0.057	0.001	0.033	0.090
Academic self-efficacy → cost → career preparation behavior	0.247	0.247	0.001	0.209	0.292

### Serial mediation effects within SEM

To ensure methodological consistency with the primary structural model analysis, the serial mediation pathways (Academic Self-Efficacy → Value → Cost → Outcomes) were examined within the SEM framework using AMOS 25.0. A bootstrapping procedure with 5,000 resamples and bias-corrected 95% confidence intervals (CI) was employed to test the significance of the indirect effects.

The results confirmed the existence of significant serial mediation effects for both outcome variables. As shown in Table 7, for Academic Performance (GPA), the specific indirect effect of the serial pathway (Academic Self-Efficacy → Value → Cost → GPA) was significant [β = 0.025, 95% CI (0.018, 0.036), *p* < 0.001]. Additionally, the parallel mediating paths via Value (β = 0.042, *p* < 0.001) and Cost (β = 0.109, *p* < 0.001) were also significant.

**Table 7:** Mediation effects for academic performance (GPA).

**Path**	**Estimate**	**95% CI**		***P***
		**Lower**	**Upper**	
Academic self-efficacy → value → academic performance (GPA)	0.042	0.021	0.068	^***^
Academic self-efficacy → cost → academic performance (GPA)	0.109	0.079	0.144	^***^
Academic self-efficacy → value → cost → academic performance (GPA) (Serial)	0.025	0.018	0.036	^***^
Total indirect effect	0.176	0.14	0.219	^***^

^***^p < 0.001. Data obtained via bootstrapping with 5,000 resamples in AMOS.

Similarly, as presented in Table 8, regarding Career Preparation Behavior, the serial mediation pathway (Academic Self-Efficacy → Value → Cost → Career Preparation Behavior) was significant [β = 0.036, 95% CI (0.026, 0.046), *p* < 0.001]. The independent mediating paths via Value (β = 0.046, *p* < 0.001) and Cost (β = 0.153, *p* < 0.001) were also confirmed.

**Table 8:** Mediation effects for career preparation behavior.

**Path**	**Estimate**	**95% CI**		***P***
		**Lower**	**Upper**	
Academic self-efficacy → value → career preparation behavior	0.046	0.025	0.07	^***^
Academic self-efficacy → cost → career preparation behavior	0.153	0.127	0.185	^***^
Academic self-efficacy → value → cost → career preparation behavior (Serial)	0.036	0.026	0.046	^***^
Total indirect effect	0.235	0.202	0.273	^***^

^***^p < 0.001. Data obtained via bootstrapping with 5,000 resamples in AMOS.

These findings support the hypothesized chain of influence where academic self-efficacy enhances perceived task value, which in turn reduces perceived costs, ultimately leading to improved academic performance and career preparation behavior.

## Discussion

Grounded in social cognitive and expectancy-value theories, this study investigated a motivational process model to understand how academic self-efficacy is associated with academic and career outcomes among students in Chinese private universities—a context often characterized by resource constraints. We proposed and tested the roles of value and cost as parallel and sequential mediators. The findings not only support the hypothesized model but also highlight the salience of cost perceptions in this particular setting, offering a nuanced view of student motivation under pressure.

### Direct effects

Academic self-efficacy exhibited a positive association with value and a negative association with cost, supporting the view that confident students are more likely to appraise academic tasks as meaningful and less burdensome (Wigfield and Eccles, 2000; Eccles and Wigfield, 2002). Moreover, self-efficacy was positively linked to both GPA and career preparation behavior, reaffirming its role in fostering goal-directed engagement (Bandura, 1997; Kim and Ra, 2022). This is particularly relevant in China's context, where academic success is tightly linked to future career prospects, especially under the pressure of high-stakes examinations and rising labor market expectations.

Perceived value contributed positively to both GPA and career preparation behavior, suggesting that when students recognize the usefulness or personal relevance of academic tasks, they are more likely to engage both academically and vocationally (Edwards and Dai, 2023). In contrast, cost, particularly emotional and opportunity-related burdens, was negatively associated with both outcomes, indicating its suppressive role in student motivation (Jiang Z. et al., 2020).

### Mediating and sequential mechanisms

The analysis revealed three key patterns consistent with theoretical mediation pathways. First, the association between self-efficacy and both GPA and career preparation behavior was partially accounted for by value, suggesting that competence beliefs may be linked to higher perceived task importance, which in turn is associated with greater engagement. Second, the negative association between self-efficacy and outcomes was partially explained by cost, implying that lower perceived self-efficacy is linked to higher costs, which in turn correlates with poorer outcomes. Third, a sequential pattern (self-efficacy → value → cost → outcomes) was identified, consistent with the notion that value and cost may sequentially transmit the influence of self-efficacy beliefs. While these structural relationships are robust within the tested model, they represent theoretical pathways that require longitudinal or experimental validation to establish directionality and causation.

### Comparison with previous research

The heightened mediating role of cost in our model, compared to findings from more resource-abundant settings (e.g., Jiang Y. et al., 2020), underscores the contextual nature of motivation. For students in private institutions facing reputational and resource challenges, the opportunity costs (e.g., time spent on academics vs. gaining marketable skills) and emotional costs (e.g., anxiety about future competitiveness) may be disproportionately salient. This suggests that theoretical models of motivation must account for institutional context, as the weight of various motivational factors may shift. Furthermore, the identified sequential pattern (self-efficacy → value → cost → outcomes) theoretically extends Flake et al. (2015) expectancy-value-cost model by proposing a motivational sequence in which value is associated with subsequent lower cost.

### Theoretical and practical implications

Theoretically, this study makes several contributions. First, by jointly modeling value and cost as parallel mediators, it reinforces the multidimensionality of motivation within expectancy-value theory, illustrating how both enhancing (value) and suppressing (cost) mechanisms concurrently operate. Second, the supported sequential pathway (self-efficacy → value → cost → outcomes) provides a dynamic, process-oriented extension to Flake et al. (2015) expectancy-value-cost model, suggesting that the appraisal of task meaningfulness may precede and influence the assessment of associated burdens. Finally, the heightened role of cost observed in our sample underscores the contextual sensitivity of motivational models, affirming that theoretical frameworks must account for institutional and resource constraints to fully explain student engagement in non-elite educational settings.

The findings offer direct guidance for educational practice, pointing to three interdependent intervention targets derived from our model. To cultivate academic self-efficacy, which serves as the model's primary antecedent, institutions should design curricula and advising that provide structured mastery experiences, scaffolded goal-setting, and constructive feedback. To enhance value, which mediated self-efficacy's positive effects, educators must explicitly link course content to vocational skills and future careers, thereby increasing the perceived usefulness and relevance of academic work. To mitigate costs, which emerged as a strong negative predictor in our context, support systems should focus on reducing psychological and logistical burdens through peer mentoring, stress-management resources, and clear guidance on navigating academic and career pathways. By addressing these three core constructs in tandem, interventions can more effectively foster both academic achievement and proactive career preparation.

## Limitations and future research

Despite its contributions, this study presents several limitations. First, the cross-sectional design restricts causal inference; while associations among self-efficacy, value, cost, and outcomes are modeled, the directionality of effects remains tentative. Longitudinal studies are needed to assess temporal precedence and mediation pathways.

Second, reliance on self-report data introduces potential biases such as common method variance and social desirability, particularly concerning students' reports of career-related behaviors. Although Harman's single-factor test did not indicate severe common method bias, future studies could incorporate objective or multi-source data.

Third, the current model includes only value and cost as mediators. Additional motivational constructs (e.g., outcome expectancy, intrinsic motivation) and contextual factors (e.g., peer influence, teacher support) may further explain students' academic and career preparation outcomes.

Fourth, the sample was limited to Chinese undergraduates, which may constrain generalizability. Cultural norms may shape perceptions of cost and the motivational salience of academic tasks; cross-cultural comparative studies are warranted.

Future research should address these limitations by (1) adopting longitudinal or mixed-method designs to test causal mechanisms and enrich interpretation; (2) exploring diverse student populations to assess model applicability across sociocultural contexts; (3) extending the model to include additional mediators and potential moderators such as gender or institutional type; and (4) investigating students' subjective experiences of cost using qualitative methods, to design targeted interventions that mitigate psychological burden and enhance motivation.

## Data Availability

The raw data supporting the conclusions of this article will be made available by the authors, without undue reservation.
